# An Experimental Test of the Persuasive Effect of Source Similarity in Narrative and Nonnarrative Health Blogs

**DOI:** 10.2196/jmir.2386

**Published:** 2013-07-25

**Authors:** Amy Shirong Lu

**Affiliations:** ^1^School of CommunicationDepartment of Communication StudiesNorthwestern UniversityEvanson, ILUnited States

**Keywords:** source similarity, tailoring, personalization, customization, narrative, blog, social media, persuasion, physical activity, health communication, health promotion

## Abstract

**Background:**

Blogs, or websites containing online personal journals, are a form of popular personal communication with immense potential for health promotion.

**Objective:**

Narratives are stories with a beginning, middle, and end that provide information about the characters and plot. Source similarity refers to the degree to which the message source and each recipient are alike with respect to certain attributes. Narratives and source similarity have seldom been examined in tandem as strategies for health persuasion. Personal health blogs provide a suitable platform for such an investigation. This study examined the persuasive effects of message type and source similarity on participants’ intentions to adopt a specific health behavior (running for exercise).

**Methods:**

A total of 150 participants were randomly assigned to conditions (n=25 per condition) in a completely crossed, 2 (message type: narrative and nonnarrative) × 3 (source similarity: no similarity, non-health-related similarity, and health-related similarity) between-subjects experiment. First, in an online questionnaire, participants provided personal information in 42 categories and rated the relatedness of each category to running and then completed pretest measures of the dependent variables. Based on their responses, 150 personal health blogs were created. Two weeks later, the initial participants read the blog created with their personal characteristics and completed a questionnaire online.

**Results:**

The source similarity effect was stronger in nonnarrative than narrative blogs. When the blogs were nonnarrative, those with health-related similarities were more persuasive than those with non-health-related similarities. Narrative blogs generated more positive thoughts and stronger blogger identification than nonnarrative blogs.

**Conclusions:**

Health-related source similarity is key for persuasive health communication, especially when the messages are nonnarrative.

## Introduction

### Background

Almost half of adult American bloggers have posted about health [1]. In addition to affecting readers’ health knowledge [2], blogs may also influence their intentions to engage in future healthy behaviors [3]. The existing health blogs could be a rich resource for health behavior promotion for both active participants and lurkers [4].

Some kinds of health blogs may be more effective than others. Suppose a young man has been recently diagnosed with Type 2 diabetes. His doctor says that the diabetes could be managed if he adopts a healthy diet. The doctor also recommends a smartphone app that, after collecting personal information, automatically retrieves blogs written by other bloggers who are making healthier diet transitions. Suppose the app would select a blog for each reader, would the blog message in the form of personal stories (narrative) or step-by-step instructions (didactic) be more helpful? What kind of blogger would be the most effective in helping this young man achieve his goals? A blogger who uses the same brand of laptop (source similarity not related to the health topic) or a blogger with the same kind of food allergy (source similarity related to the health topic)?

The general research question addressed in this study is what blog features enhance the effectiveness of blogs for delivering persuasive health messages? Two specific blog features were examined. The first feature is whether the blog message is narrative or nonnarrative. A narrative consists of “any cohesive and coherent story with an identifiable beginning, middle, and end that provides information about scene, characters, and conflict, raises unanswered questions or unresolved conflict; and provides resolution” [5], whereas a nonnarrative consists of “arguments, reasoning, claims, and so forth” [6] that are “overtly persuasive messages” [7]. Narratives, or stories, are more available as carriers of personal health information [8-10]. Increasing evidence suggests that narratives are an important persuasion tool with unique capacities [11] and are processed differently than nonnarratives [12].

The second feature is the nature of the blogger’s similarity (source similarity) with the user (or blog visitor). Similarity, which refers to the degree to which 2 persons are alike with respect to certain attributes [13], has been found to be an important dimension in persuasion [14-18]. Unrelated similarities may help gain rapport, but not credibility [19]. In this study, similarity refers to matching blogger characteristics to each individual reader. The role of similarity is treated as an empirical question. Specifically, the experimental blogs differed in whether the blogger was depicted as similar to the user in health-related characteristics (or characteristics relevant to the particular health behavior of interest), or as similar in non-health-related characteristics (or characteristics irrelevant to the particular health behavior of interest).

These 2 blog features, the use of narratives and the type of source similarity, were expected to have both independent and joint effects on the users. The interplay of the 2 was empirically explored in personal health blogs as a form of health communication. The specific hypotheses are discussed in the following section.

### Hypotheses

#### Message Type: Narrative vs Nonnarrative

Previous efforts comparing the persuasive effects of narratives and nonnarratives have yielded equivocal findings [20,21]. One possible explanation for such mixed findings is that there have been no standard criteria to compare narratives and nonnarratives. Another important explanation is that many of the studies relied on the dual-processing models of persuasion [22,23], which may not be the most appropriate for studying narratives.

Transportation, along with absorption and engagement, are used interchangeably to indicate people’s immersive experience with narratives [24]. A unique mental process, an integrative melding of attention, imagery, and feeling in which a reader becomes absorbed in a narrative world [6], transportation has been identified as a unique persuasive mechanism of narratives as it engages people in behavioral rehearsal [25], appears less overtly persuasive [26], reduces counterargument [27], and enhances message recall [11]. Attitudes formed or changed via transportation are strong, persistent over time, and resistant to counterargument, even though they may not be centrally processed [28]. Blogs, by virtue of their relative personal communication form (almost diary-like) naturally lend themselves to the presentation of personal stories. Therefore, our first hypothesis is that narrative blogs will be more persuasive than nonnarrative blogs.

#### Source Similarity: Health-Related, Non-Health-Related, None

Similarity, or the degree to which 2 entities are alike with respect to certain attributes [13], appears to influence persuasive outcomes indirectly by affecting the receiver’s liking and the receiver’s perception of the credibility of the source [29]. Similarity must be studied in conjunction with relatedness [30] because source similarity on unrelated characteristics may help gain rapport, but not credibility [19]. A blog containing non-health-related similarities might plausibly make the blogger more liked because apparent attitudinal similarity increases liking, whereas the health-related similarities might plausibly increase both liking and credibility [29]. Source similarity, therefore, should be about characteristics related to the theme of the message [31,32] to maximize the persuasive outcome. Therefore, our second hypothesis is that (1) the blogs with health-related similarities will be more persuasive than those with non-health-related similarities, (2) the blogs with health-related similarities will be more persuasive than those with no similarities, and (3) the blogs with non-health-related similarities will be more persuasive than those with no similarities.

#### Interaction of Message Type and Source Similarity

When the blogs are nonnarrative, source similarity should increase personal relevance, elevating the reader’s motivation and ability to process the message [22,33]. When the blogs are narrative, source similarity can make it easier for readers to connect or to identify with characters, thus facilitating transportation, which could lead to more persuasion [34]. Therefore, the interaction between message type and source similarity will be treated as an empirical research question: in which message type will source similarity have a stronger persuasion effect?

## Methods

### Participants

The experimental procedure had been refined by a prior independent pilot study with 30 participants. A total of 300 undergraduate students (150 male and 150 female) from University of North Carolina at Chapel Hill, Chapel Hill, North Carolina, were invited to participate with a completely cross-balanced, 2 (message type: narrative and nonnarrative) × 3 (source similarity: no similarity, non-health-related similarity, and health-related similarity) between-subjects factorial design. One-half of participants were recruited from 15 classes. One-third were recruited through advertisements posted on the bulletin boards of 3 departments, 3 libraries, and 3 student organizations on campus. The rest were solicited directly from a campus dining hall.

### Pretest Questionnaire

The 300 participants were first asked to fill out an online questionnaire, which helped to identify a health behavior most were not doing regularly, but were planning to start or do more regularly; to collect personal information and perceived relatedness of the personal information regarding the health behavior for the subsequent procedures; and to obtain baseline dependent measures.

Three health behaviors (running, yoga, and eating 5 servings of fruits and vegetables per day) were listed as potential options according to recent college health research [35,36]. Each health behavior was feasible to carry out regularly (≥4 times per week) for a typical undergraduate. To ensure that other highly desired healthy behavior was not excluded, participants were also allowed to indicate a behavior they planned to engage in that was not on the list.

Two criteria were used to select the focal health behavior: only a few students should be engaged in the behavior initially, but most should show a moderate level of interest in the behavior in the future. Of the 300 students, 222 (74.0%) completed the pretest questionnaire. Fifty (22.5%) were already regular runners and 185 (83.3%) showed some interest in starting to run or running more regularly. Although 5 (2.3%) were regular yoga practitioners, only 137 (61.7%) expressed interest in practicing yoga in the future. Because 84 (37.8%) participants reported eating 5 servings of fruits and vegetables per day, too few would be eligible for behavior change. Another 61 (27.5%) of the participants mentioned a total of 14 other health behaviors. Thus, of all potential health behaviors, running for exercise best met the selection criteria; therefore, it was used in the main experiment.

Based on extensive reading of running books and magazines and interviews with amateur and professional runners and exercise scientists, 21 characteristics related to running (eg, ideal frequency of running per week) and 21 not related to running (eg, favorite T-shirt color) were identified. Data saturation was reached. Participants were instructed to provide personal information about themselves in all of the 42 categories and rated the relatedness of each category to running based on a 7-point scale (1=extremely unrelated; 7=extremely related). Their answers were carefully screened. If a participant answered questions generally rather than specifically (ie, answered “What is your favorite movie” with “I like many kinds of movies,” rather than a specific movie title), the answer was not considered unique personal information and was not used. After all usable answers had been identified, those that had been rated by the participant 4 or greater on the related to running scale were considered related and those less than 4 were considered unrelated.

Pretest measures of the dependent variables (described subsequently) were also collected.

### Blog Prototype Creation

**Figure 1:**
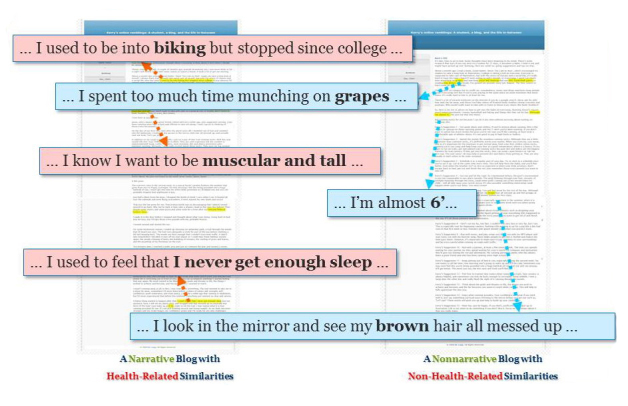
Blog construction.

Two single-page blog prototypes (narrative and nonnarrative) were created with the help of 2 professional writers and psychologists and edited to the same length (1293 words). Each prototype either stayed alone as the generic narrative and nonnarrative with no similarity blog entries or allowed the insertion of the blogger’s information in up to 42 exclusive categories without invoking logical errors or inconsistencies. However, interviews with another group of 5 undergraduate and graduate students (Question: How many personal characteristics do you think should be embedded into these blog prototypes to make the participants realize the source similarities but not get overwhelmed or suspicious? Answers were coded into numbers and averaged) helped determine that no more than 6 personal characteristics should be embedded in a blog to avoid suspicion and potential negative reaction to the blog. A unique or obscure personal characteristic (eg, “Mack Rice’s Three People in Love [1968]” as a favorite song) was inserted as a categorical description (eg, “some early style funky music in the 1960s”) instead of a verbatim match to prevent an incredulity reaction. Six personal characteristics related or unrelated to running identified in the pretest were then randomly selected for each participant and were inserted in 1 of the 2 blog prototypes (Figure 1). The no-similarity blogs were adopted directly from the generic prototypes and did not have unique personal characteristics inserted.

**Figure 2:**
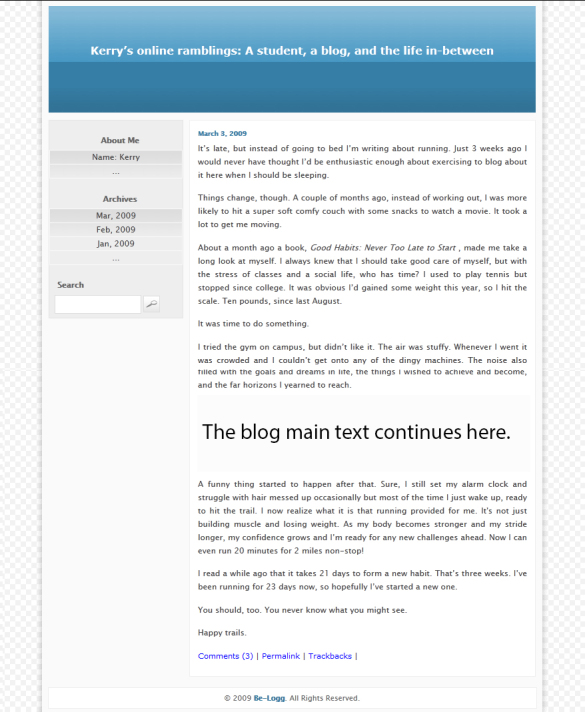
Experimental blog.

The blog was titled “Kerry’s online ramblings: A student, a blog, and the life in-between.” The blogger was named Kerry to avoid a gender confound because “Kerry” was rated to be the most gender-neutral name among an independent pilot study with 30 participants (mean 2.22, SD 0.71; with 1=male; 2=both; 3=female). As shown in Figure 2, the blog had 3 parts. The left panel showed the blogger’s name and basic information. The right panel showed a calendar. The middle panel contained the main experimental blogs.

In the narrative blog prototype, the blogger described how he or she decided to start running (beginning), how difficulties were overcome (development), and ended with a dramatic encounter with a deer during a morning run (climax/ending): “...I ended up choosing an unfamiliar path...Just as I entered the lane and turned a corner...a large deer wandered onto the path. We looked at each other for a brief moment before it nimbly turned around and bounded gracefully off through the woods and disappeared...” In the nonnarrative blog prototype, the blogger provided a total of 15 pieces of didactic suggestions arranged as bullet points on why people should run. For example, the deer encounter appeared as: “Suggestion 12—Feel free to explore new routes when running on trails. New scenery is always helpful, and sometimes you may be lucky enough to encounter some wildlife, such as a deer, like I did.”

The 222 participants were randomly assigned to one of the 6 experimental conditions, with 37 persons per condition. Accordingly, 222 personal blogs were created and uploaded to a Web server. All blogs were edited to be approximately the same length. Design and layout (eg, blogger name, basic information, calendar) were identical across conditions except for manipulation of the 2 independent variables (message type and source similarity) in the main experimental blog section. Each participant saw 1 blog post for this study. The commenting function was disabled for control purpose.

### Main Experimental Procedures

At least 2 weeks after the pretest, students participated in the experimental session. To weaken the association between the pretests and posttests, the 2 sessions were promoted with different titles and the questionnaires had different layout designs. In the experiment session, each participant sat at a computer and was instructed to read and sign the consent form. Participants in the health-related similarity and non-health-related similarity conditions saw a (narrative or nonnarrative) blog matched on his or her earlier pretest responses and those in the no-similarity condition saw a generic (narrative or nonnarrative) blog. The time each participant spent reading the blog was recorded by an embedded Web app.

**Figure 3:**
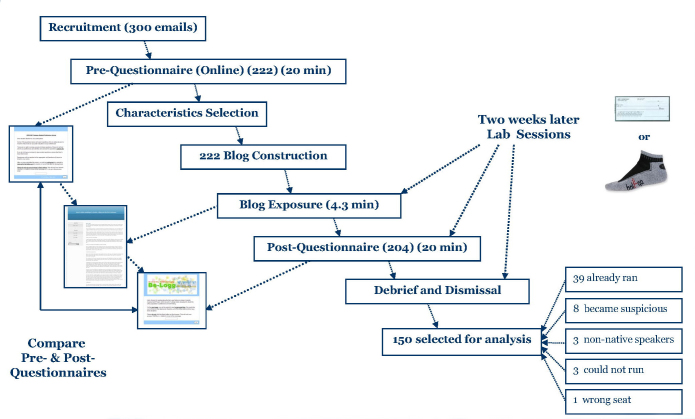
Experimental procedure.

Of the 222 participants who participated in the pretest, 204 (91.9%) completed the posttest. Of the 204 participants, 39 (19%) identified themselves as regular runners who ran at least 30 minutes 4 times a week; 8 (4%) figured out that their answers to the pretest were used to create the blogs and guessed both projects were related; 3 (2%) were nonnative English speakers; 3 (2%) wrote explicitly that they did not want to run at all because of health conditions (depression, fracture, and paralysis); and 1 (0.5%) accidentally completed the survey assigned to another student by sitting at the wrong computer. These 54 (27%) students were excluded from the analysis, resulting in a sample of 150 participants, with 25 participants per condition. Figure 3 provides a flowchart for the complete experimental procedure.

### Dependent Measures

The primary outcome variables were 3 independent measures assessing participants’ intention to run for exercise based on established measures and consultation with runners and exercise scientists. One was a single-item measure asking participants the likelihood of running in the near future on a unipolar scale [37]: “Please indicate the likelihood you would start running for 30+ minutes for 4+ times per week in the near future (1=not at all likely; 7=extremely likely).” The second asked about the participant’s intended running duration: “If you start running in the near future, how long would you like to do it every time? __ Minutes.” The third was a behavioral measure: running-related gift selection. Before leaving the computer laboratory, each participant was offered 2 compensation options: a US $10 check or 2 pairs of running socks. Both options were perceived by a separate group of students as worth the same monetary value. The socks were unisex, of different sizes, popular among runners, specifically designed for trail running, and available only in professional running stores. Because the blog advocated running for exercise and the blogger had been running on a trail, participants’ choosing a form of compensation specifically designed for the advocated health behavior could serve as an additional indicator of the context-specific persuasion outcome.

### Psychological States as Potential Mediating Variables

The main goal was to explore how message type and source similarity affected people’s response to persuasive messages; therefore, hypotheses were tested by relying on the blog’s intrinsic features and manipulation checks were not conducted [38]. Three variables (narrative transportation, source similarity, and relatedness) were assessed with Likert-style 7-point scales (1=strongly disagree; 7=strongly agree) and were summed and averaged as participants’ psychological states in response to the message manipulation. Narrative transportation was assessed by using Green and Brock’s [12] 11-item scale, which demonstrated good internal consistency (α=.88). Sample items included: (1) I want to learn how the blog would end, and (2) I can picture myself in the blog. Source similarity was assessed with a 2-item measure adapted from customization research [39], which exhibited a strong correlation (*r*=0.80, *P*<.001): (1) this blog targeted me as a unique individual, and (2) this blog was personalized according to my interests. Relatedness was assessed with a 2-item measure: “The similarities between Kerry and me are…” and “the characteristics shared by Kerry and me are...” related to whether I will begin running 30+ minutes for 4+ times per week in the near future. The 2 items were strongly related (*r*=0.77, *P*<.001).

### Blog Processing and Involvement Variables

Six theoretically relevant variables were explored. Two assessed readers’ blog processing (number of meaning units and thought valence). Meaning units refers to a collection of words related to 1 central meaning, also known as an idea unit [40]. In the posttest questionnaire, participants were asked to list all of the thoughts they had while reading the blog [41]. Two native-English speakers unaware of the experimental purpose served as coders to count the number of relevant meaning units. The coders also coded the valence of each relevant thought (-1 for negative; 0 for neutral; and +1 for positive). The valence scores of each thought were aggregated into a valence scale for each participant.

Four variables addressed readers’ involvement with the blogger. All were assessed on 7-point Likert scales (1=strongly disagree; 7=strongly disagree). Perceived source credibility was assessed with a 6-item scale adapted from Metzger and colleagues [42] and showed good internal consistency (α=.93) (sample items are “I trust the blog Kerry posted” and “Kerry is credible”). Interpersonal attraction was assessed with a 10-item scale adapted from McCroskey and McCain [43] that achieved adequate internal consistency (α=.78) (sample items are “I think Kerry could be a friend of mine” and “I would like to have a friendly chat with Kerry”). Identification with the blogger was measured with 7 items selected from Eyal and Rubin’s [44] 10-item scale that achieved strong internal consistency (α=.90) (sample items are “when I was reading the blog, I imagined myself doing the same things Kerry was doing” and “I really felt as if I was Kerry who was running”). Parasocial interaction was assessed with Rubin’s [45] scale (α=.90) (sample items are “Kerry makes me feel comfortable, like I’m with a friend” and “I look forward to reading Kerry’s blog when more are posted”). Although formal hypotheses were not proposed for the involvement variables, they proved valuable in understanding the process and consequence of the interaction of the message type and source similarity.

## Results

### Preliminary Analyses

The 150 students came from 33 academic majors across campus (17.3% freshmen, 31.3% sophomores, 20% juniors, and 31.3% seniors). Sixty-eight (45.3%) were male and 82 (55.7%) were female. The mean age was 21.2 years (SD 2.3). They spent 4.3 hours (SD 1.6) online per day. The 150 blogs were carefully edited to be approximately 1300 words across conditions (mean 1316, SD 22). The health-related similarity blogs (mean 1342, SD 12) were longer than the non-related similarity blogs (mean 1314, SD 9), which were longer than the no-similarity, or generic blogs (mean 1293, SD 0; *P*<.001). There were no significant word count differences between the narrative and nonnarrative blogs. The average reading time was 256.8 seconds (SD 100.7), which did not differ across conditions (*P*=.20).

Analyses of variance (ANOVAs) were conducted on both likelihood of running and intended running duration by message type and source similarities. Participants across conditions did not differ on the pretest measures, and there was no significant gender or age difference. Paired sample *t* tests indicated that both posttest measures had significantly increased from the pretest (*P*<.001).

Although each blog with similar personal characteristics (health-related and non-health-related) differed in specific personal characteristics depending on each individual’s ratings, according to the cumulative relatedness ratings across the 42 categories, 5 characteristics, including free time, exercise frequency, ideal body shape, lack of sleep, and current exercise were rated as the overall most related, whereas favorite bookstore, hair color, favorite book, favorite T-shirt color, and favorite news site were rated as the overall least related.

A 2 (narrative and nonnarrative) × 3 (no similarity, non-health-related similarity, and health-related similarity) ANOVA indicated that people responded differently to narrative and nonnarrative blogs (*F*_1,144_=13.46, *P*<.001, partial η^2^=0.09). Transportation was significantly higher in the narrative conditions (mean 4.58, SD 0.96) than in the nonnarrative conditions (mean 3.93, SD 1.19). Neither the main effect or interaction for source similarity on transportation was significant.

Two 2-way ANOVAs were conducted to explore participants’ response to source similarity. The first 2 (narrative and nonnarrative) × 3 (no similarity, non-health-related similarity, and health-related similarity) ANOVA showed a significant main effect for the source similarity (*F*_1,144_=7.69, *P*=.001, partial η^2^= 0.10). Post hoc Tukey honestly significant difference (HSD) comparisons indicated that the mean scores in health-related (mean 3.84, SD 1.75) and non-health-related similarity conditions (mean 4.00, SD 1.78) were significantly higher on the source similarity scale than those with no similarities (mean 2.77, SD 1.55; *P*=.001). There was no significant difference between the 2 similarity conditions (*P*=.89) or any significant main effect or interaction for message type on source similarity type (*F*_1,144_=1.06, *P*=.31). The second 2 (non-health-related similarity and health-related similarity) × 2 (narrative and nonnarrative) ANOVA revealed a significant main effect on relatedness for both similarity conditions (*F*_1,96_=4.98, *P*=.03, partial η^2^=.05). The relatedness score of participants in health-related similarity (mean 4.18, SD 1.12) was significantly higher than those of participants in the non-health-related similarity (mean 3.66, SD 1.19; *P*=.001). Neither the main effect nor the interaction for message type on relatedness were significant.

### Testing of Hypotheses

To test the hypotheses, two 2 (message type: narrative and nonnarrative) × 3 (source similarity: no similarity, non-health-related similarity, and health-related similarity) analyses of covariance (ANCOVAs) were performed on the likelihood of running and the intended running duration with the posttest measures as dependent variables and the pretest measures as covariates. In all ANCOVAs, the pretest measures were significant and were not reported individually. For health-related gift selection, the only categorical outcome, full and conditional cross-tabulations were performed.

The first hypothesis predicted that narrative blogs would be more persuasive than nonnarrative blogs. This was not supported by the likelihood of running or the health-related gift selection (chi-square [χ^2^_1_] =1.43, *P*=.23). The 2 × 3 ANCOVAs only revealed a non-significant effect for the intended running duration (*F*_1,143_=3.30, *P*=.07, partial η^2^=0.02) for narratives (mean 32.60, SD 10.57) over nonnarratives (mean 29.27, SE 9.10). Therefore, the first hypothesis was not supported.

**Figure 4:**
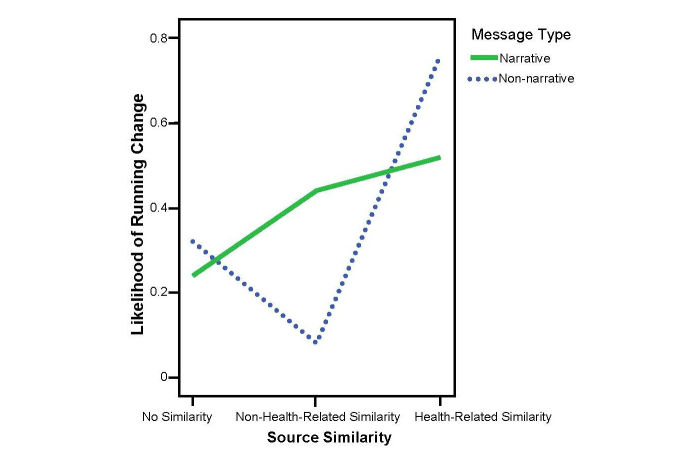
Interaction of message type and source similarity on likelihood of running change.

The second hypothesis predicted that blogs with health-related similarities would be more persuasive than those with non-health-related similarities and those with no similarities. For the likelihood of running, source similarity was not significant (*P*=.15). Post hoc comparisons showed no differences in source similarity across the narrative conditions (*P*=.98). In the nonnarrative conditions, however, the non-health-related similarity condition (mean 3.04, SD 1.51) was significantly lower than that of the health-related similarity condition (mean 4.32, SD 1.60; *P*=.01). Although the non-health-related similarity condition was lower than that of the no similarity (mean 4.12, SD=1.56; *P*=.06), it did not meet statistical significance. There was no difference between the related and the no-similarity conditions (*P*=.49). See Figure 4 for the change scores across conditions.

**Figure 5:**
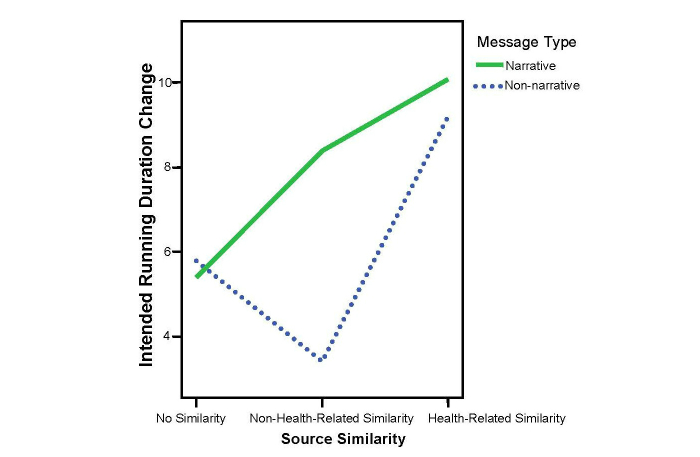
Interaction of message type and source similarity on intended running duration change.

For intended running duration, ANCOVA showed a significant effect for source similarity (*F*_2,143_=3.17, *P*=.04, partial η^2^=0.04). Post hoc comparisons indicated a similar pattern: the effect was also primarily due to the non-health-related similarity condition (mean 25, SD 8.17), which was lower than the no-similarity generic condition (mean 29.80, SD 8.48; *P*=.05) and significantly lower than the health-related similarity condition (mean 33, SD 9.13; *P*=.002). The no-similarity condition generated a shorter intended running duration than the health-related similarity condition, although it was not statistically significant (*P*=.09). See Figure 5 for the change scores across conditions.

**Figure 6:**
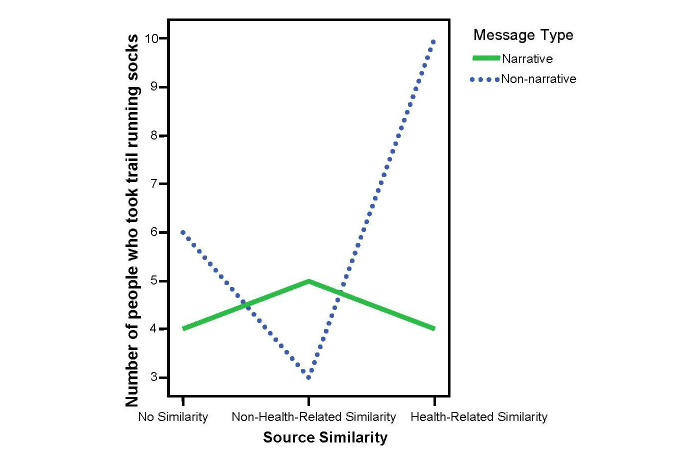
Interaction of message type and source similarity on health-related gift selection.

For health-related gift selection, 32 (21.3%) participants chose to get the socks instead of the check. The chi-square test was not significant (χ^2^_2_ =2.23, *P*=.33). Analyses of the nonnarrative data showed that more participants in the health-related similarity conditions chose the socks (n=10) than the non-health-related (n=3) and the no-similarity conditions (n=6), (*P*=.02 and *P*=.04, respectively). See Figure 6 for the scores across conditions.

Thus, the first part of the second hypothesis, the blogs with health-related similarities will be more persuasive than the blogs with non-health-related similarities, was supported when the message was nonnarrative. The second part of the second hypothesis, the blogs with health-related similarities will be more persuasive than the blogs with no similarities, was partially supported by health-related gift selection when the message was nonnarrative. The first hypothesis, the narrative blogs will be more persuasive than nonnarrative blogs, and the third part of the second hypothesis, the blogs with non-health-related similarities will be more persuasive than blogs with no similarities, were not supported.

To answer the research question, in which message type will source similarity have a stronger persuasion effect, the factor of message type (2) × source similarity (3) was examined and found to be not significant for the likelihood of running and the intended running duration (*P*=.17 and *P*=.73, respectively). A 2 (narrative and nonnarrative) × 2 (non-health-related similarity and health-related similarity) ANCOVA, however, provided some evidence: the difference between health-related and non-health-related similarity for nonnarratives was bigger than that for narratives (*F*_1,95_=3.38, *P*=.06, partial η^2^= 0.03) for the likelihood of running. In other words, the effect of whether the similarity was health-related was more pronounced in the nonnarrative conditions than in the narrative conditions. This result, however, did not meet statistical significance. For the health-related gift selection, the differences appeared within source similarity types under nonnarrative conditions (see the second hypothesis and Figure 6), but not in narrative conditions (χ^2^_1_<0.2). So source similarity seems to have a stronger persuasive effect in the nonnarrative context than in the narrative context.

### Blog Processing Variables

Analyses of the number of meaning units and thought valence suggested that readers processed narratives and nonnarratives differently. Although the 2 (narrative and nonnarrative) × 2 (no similarity and similarity including non-health-related and health-related combined) ANOVA was not significant, the post hoc multiple tests showed that for blogs with source similarities, health-related or not, nonnarratives (mean 10.50, SD 6.17) produced more thoughts than narratives (mean 8.52, SD 4.32, *P*=.06) although the result did not meet statistical significance. Readers also had more positive thoughts about narratives (mean 1.10, SD 4.27) than nonnarratives (mean –0.89, SD 5.53; *F*_1,144_=6.18; *P*=.01; partial η^2^=0.041).

### Involvement Measures

All involvement measures were analyzed by 2 (narrative and nonnarrative) × 3 (no similarity, non-health-related similarity, and health-related similarity) ANOVAs except for interpersonal attraction, which was analyzed in a 2 (narrative and nonnarrative) × 2 (no similarity and similarity including both health-related and non-health-related) ANOVA. There was a main effect for source similarity, *F*_1,144_=3.89, *P*=.05, partial η^2^= 0.03, for perceived source credibility: bloggers with non-health-related similarities were perceived as less credible (mean 4.70, SD 1.18) than bloggers with health-related similarities (mean 5.13, SD 0.65). Source similarity had an effect (*F*_1,146_=3.22, *P*=.07, partial η^2^= 0.02) on interpersonal attraction: blogs with similarities (mean 4.60, SD 0.67), health-related or non-health-related, were perceived as more attractive than bloggers with no similarities (mean 4.42, SD 0.69) although this did not meet statistical significance.

Readers showed more identification with bloggers of narrative messages (mean 4.20, SD 1.38) than of nonnarrative messages (mean 3.70, SD 1.40; *F*_1,144_=7.43, *P*=.007, partial η^2^= 0.05). They also showed a higher level of parasocial interaction with bloggers of narrative messages (mean 4.43, SD 1.23) than of nonnarrative messages (mean 4.14, SD 1.29; *F*_1,144_=4.7; *P*=.03; partial η^2^= 0.03).

Each of the potential mediating and involvement variables was included as a control in the hypotheses testing 2 × 3 ANCOVA models. Although they were significantly correlated with the outcome variables, and none of the previous significant effects were eliminated, they were not significant in the models, suggesting that these variables were not mediating the effect of message type and source similarity on the dependent variables. Gender was included as a third independent variable in all of the hypotheses testing ANCOVA models with no significant main effects or interactions.

In summary, the source similarity made much more difference in nonnarrative messages than in narrative messages. Nonnarrative blogs were much more persuasive if they contained health-related similarities than if they contain non-health-related similarities. When the blogs were nonnarrative, source similarity led to an increase in the number of thought meaning units. Although bloggers with similarities were perceived to be more attractive than bloggers with no similarities, those with health-related similarities were perceived to be more credible than those with non-health-related similarities. Compared with nonnarrative blogs, narrative blogs elicited more positive thoughts. Readers of narrative blogs identified more with the bloggers and were more likely to feel some parasocial interaction with them than readers of nonnarrative blogs. The source similarity, however, does not make much of a difference to the persuasive outcome narrative blogs.

## Discussion

### Principal Findings

This study is one of the first systematic empirical examinations of the interplay of message type and source similarity in personal health blogs. Significant differences in healthy behavior intention were detected depending on the kind of source similarity in the nonnarrative health blogs, but not in the narrative health blogs: blogs with health-related similarities were more persuasive than blogs with non-health-related similarities.

Why would source similarity have a stronger persuasion effect in nonnarrative messages than in narrative messages? In the narrative context, although source similarity may make it easier for readers to connect with characters, thus facilitating transportation [46], source similarity, however, is not necessary for transportation. A skilled writer may also make a story relevant to his readers through other elements, such as vivid descriptions and engaging plots [24]. Therefore, when blogs are engaging narratives, readers may be already involved and may not scrutinize the messages or evaluate the relatedness of source similarities as much as those nonnarrative blog readers.

In a nonnarrative context, the blogger’s credibility could play more of a role. Therefore, variables that would influence credibility (eg, health-related similarities) will correspondingly have a larger role to play in nonnarratives as compared to narratives. In the nonnarrative context, the personal health blog is more prescriptive than descriptive, which is highly personally relevant. The source similarity manipulation is both wide and deep, which has been confirmed by each recipient’s input [47-49]. Readers were less likely to treat the similarities as heuristic cues. Such personal relevance would increase readers’ motivation to process persuasive information [22] systematically rather than heuristically [33]. Some evidence existed for this explanation in that readers in both source similarity conditions had significantly more meaningful thought units than those in the no-similarity conditions. As a result of central processing, readers of nonnarrative blogs may have scrutinized the blogs with more sensitivity to the similar personal characteristics than readers of narrative messages, who were already more transported into the story.

Although the source similarity effect was stronger in the nonnarrative conditions, narratives should not be written off as unimportant. The narratives could motivate people to increase their physical activity even when the blogs were not specifically created with source similarities inserted as blogger characteristics. The narrative readers who enjoyed the running experience in the stories might imagine running longer because of their pleasant reading experience [50]. The positive thought valence for the narratives provides some support for this idea. In contrast, the nonnarratives provided only didactic instructions, which might provide a less enjoyable reading experience. The participants could have felt they were being “talked at,” which generated less positive feelings. This pattern could also be explained by psychological reactance theory [51], which posits that reactance will occur when people perceive that their freedom of choice is threatened. People may feel less threatened by narratives and, thus, less resistant to the messages as they are free to use their imaginations as they are transported into the story. In this study, the message type (narrative and nonnarrative) manipulation was based on 2 prototypes previously created without consulting with each participant’s narrative/nonnarrative preference, whereas the source similarity (no similarity, non-health-related similarity, health-related similarity) was manipulated thoroughly based on each participant’s input. If the message types could be manipulated in the same way, it is likely that a more significant difference could be observed. In fact, although the blogger in the nonnarrative blog conditions was giving a list of suggestions, the blogs were still written in the first person perspective. It also mentioned a personal experience (deer encounter), a conflict (to choose a new route or not), and a solution (yes, to choose a new route), all of which might even evoke some slight unconscious narrative processing among participants as people have an innate tendency to process information as narratives. This could also explain the lack of persuasive outcome difference between the 2 conditions.

This study demonstrated that to be effective, the source similarity “match” must be well integrated with the persuasive messages. The difference between health-related and non-health-related similarities could also be rephrased as the distance between different aspects of the self [4]. An examination of the top 5 most health-related and non-health-related similarities suggested such a pattern: most of the health-related similarities were personal characteristics (eg, exercise frequency) and the non-health-related similarities were personal preferences (eg, favorite book). Personal preferences define what a person likes whereas personal characteristics define who a person is. Therefore, source similarity should be aligned with characteristics that are central to the recipient’s self-concept.

Useful insights for health communication can be drawn. Blogs may be a useful tool for encouraging people to adopt healthy habits. In this study, just 1 exposure significantly increased both measures of behavioral intentions. Such interventions could be further integrated with social networking sites. After people provide information about themselves, they could be directed to the online virtual blogger communities that would be most helpful for them. Health communicators should be careful in selecting appropriate personal characteristics on which to create persuasive messages with similar sources. In this study, non-related source similarities could backfire and even cancel out the increased persuasion effect due to related source similarities, resulting in the lack of difference between the similarity and no-similarity conditions.

Health communicators should be extremely careful selecting appropriate characteristics on which to create customized health messages. When resources are too limited to effectively evaluate the most appropriate characteristics for customization, creating a generic transporting narrative message may be the most cost-effective solution.

In fact, a message with both narrative and nonnarrative elements may be most effective. Narratives may help reduce the initial psychological reactance to the persuasion by transporting readers into the narrative world [27]. Once the audience is on board, an appropriately created nonnarrative message with their prior input could be delivered. When done well, such practice should create the feeling of interpersonal communication [52].

### Limitations

This study has limitations. The narratives created by academic researchers are usually not as transporting as those written by professional writers. The narrative manipulation could have been more thorough and in-depth. Although the nonnarrative blogs were less prose-like and did not fully conform to the narrative definition [5], they could still retain some narrative elements or even evoke narrative processing. The all-student sample read only 1 experimental blog, and posttest measures were taken immediately after the exposure. Due to the lack of statistical power, results of several statistical comparisons did not meet statistical significance and mediation analyses were precluded. Although choosing the trail running socks might indicate the intention to run, choosing the cash prize did not necessarily indicate an absence of running intention as people may prefer the cash over the socks despite high intentions to exercise. The mix of domains (money vs a specific item) might pose potential threat to internal validity. Other than the gift selection behavior, no actual health behavior was measured. Although no student was able to correctly identify the purpose of the study, demand characteristics could be still at play. For experimental control, participants were unable to post comments on the blogs. Repeated exposure to multiple blog entries, delayed posttest measures, and user comments may result in different attitudinal and behavioral change patterns. The self-concept is highly volatile and can be easily changed by priming [53]. Health professionals could adapt to the volatility of self-concept by identifying more intrinsic and stable characteristics. The pretest questionnaires required participants to answer more than 130 questions for at least 20 minutes. Online users may be reluctant to devote so much time to providing so much personal information. Alternative plans should be devised to collect personal information without arousing suspicion or fatigue.

### Conclusions and Future Research

Future research could follow several different paths. Source similarity could and should be treated in a more fine-tuned manner. Instead of emphasizing the importance of source similarity over no similarity, different types of source similarity should be explored and compared. More studies should be conducted to identify the minimum effective number of personal characteristics necessary. Although no significant mediation effect was detected in this study, future studies could adopt relevant variables in its design to better detect the persuasive mechanism. No actual exercise data were collected in this study, which constitutes a major limitation. Future studies could incorporate objective behavior measures collected from body sensors and accelerometers. The present research should also be replicated across different media platforms among different populations. Research has shown that people also prefer attending to arguments that highlight abstract rather than concrete features when attitude objects are temporarily distant [54]. In this study, almost all source similarity aspects were concrete and only a health behavior in the near future was examined. More research is needed to explore the feasibility of creating source similarity using abstract, or higher-level, personal characteristics (eg, personality characteristics such as introversion or extroversion) for different behaviors and other behavioral outcomes. Finally, the growing recognition of culture as an important factor in health communication has the potential to contribute to the development of new and more effective message design. Although cultural values (eg, collectivism vs individualism) may not be inherently health-related, they may still influence health outcomes of the individuals and may enhance receptivity, acceptance, and salience of health messages [55].

To conclude, 2 types of messages (narrative and nonnarrative) and 3 types of source similarity (no similarity, non-health-related similarity, and health-related similarity) were empirically explored in a blog promoting the virtues of running. The results suggest that health-related source similarity is key for persuasive health communication especially when the messages are nonnarrative.

## Abbreviations

ANCOVA: analysis of covariance
ANOVA: analysis of variance
HSD: honestly significant difference
